# EPIDEMOLOGICAL, RADIOGRAPHIC AND PROGNOSTIC EVALUATION OF CHONDROBLASTOMA

**DOI:** 10.1590/1413-785220243201e283605

**Published:** 2025-04-07

**Authors:** BERNARDO LOPES CRISOSTOMO, JULIA POZZETTI DAOU, JAIRO GRECO GARCIA, MARCELO DE TOLEDO PETRILLI, DAN CARAI MAIA VIOLA, REYNALDO JESUS GARCIA

**Affiliations:** 1 Universidade de Sao Paulo, Faculdade de Medicina, Hospital das Clinicas HC-FMUSP, Departamento de Ortopedia e Traumatologia DOT, Disciplina de Ortopedia Oncológica, Sao Paulo, SP, Brazil.; 2 Hospital Israelita Albert Einstein, Grupo de Ortopedia Oncológica, São Paulo, SP, Brazil.

**Keywords:** Chondroblastoma, Bone Neoplasms, Risk Factors, Artrosis, Recurrence, Condroblastoma, Neoplasias Ósseas, Fatores de risco, Artrose, Recidiva

## Abstract

**Objective:**

To describe the clinical and imaging characteristics of chondroblastoma and identify possible factors related to joint complications.

**Method:**

This retrospective cohort study was carried out with data from the medical records of 23 patients diagnosed with chondroblastoma, subjecting them to statistical analyses.

**Result:**

In total, 19 patients were included, 12 (63.2%) of which were mean with a mean age of 13.6±3.5 year. The relation with the local dimension equaled 57.9%, higher in the apophysis of the greater trochanter: 95.2% (p<0.001). Based on imaging, 15.8% patients had an open physis; 55.6%, no damaged physeal line; 42.1%, cortical rupture; 21.1%, secondary aneurysmal bone cyst; 26.7%, violated cartilage; and all cases, medullary edema. 15.8% of cases showed local recurrence and no metastasis. Moreover, 46.7% of patients had relevant secondary osteoarthritis related to the aggressiveness of the tumor according to the Enneking classification (p= 0.041).

**Conclusion:**

The clinical outcome of chondroblastoma show no relation to age, sex, location, physeal status, or presence of calcifications or secondary aneurysmal bone cyst. Progression to secondary osteoarthritis configured the most frequent non-oncological complication and showed a direct relation with the severity of the chondroblastoma. ***Level of Evidence IV, Case Series.***

## RESUMO

**Objetivo:**

Descrever as características clinicas e imaginológicas do condroblastoma e identificar possíveis fatores relacionados a complicações articulares.

**Método:**

Estudo de coorte retrospectiva realizado utilizando-se dados de prontuários de 23 pacientes com diagnóstico de condroblastoma, submetidos a análise estatística.

**Resultado:**

Foram incluídos 19 pacientes, 12 (63,2%) do gênero masculino, idade média 13,6±3,5. A relação com a dimensão local foi de 57,9%, maior na apófise do grande trocanter com 95,2% (p<0,001). Do ponto de vista imaginológico, tiveram status fisário aberto 15,8%; 55,6% não violavam a linha fisária; rotura cortical em 42,1%, cisto ósseo aneurismático secundário em 21,1%, cartilagem violada em 26,7% e todos casos apresentaram edema medular. 15,8% dos casos apresentaram recidiva local e nenhuma metástase. 46,7% dos pacientes tiveram artrose secundaria relevante, com relação com a gravidade do tumor pela classificação de Enneking (p= 0,041).

**Conclusão:**

O desfecho clínico do condroblastoma não apresentou relação com idade, sexo, localização, status fisário, presença de calcificações ou cisto ósseo aneurismático secundário. A evolução para artrose secundaria foi a complicação não oncológica mais frequente, e mostrou relação direta com a gravidade do condroblastoma. **Nível de Evidência IV, Série de Casos.**

## INTRODUCTION

Epiphyseal chondroblastoma was first described in 1931^1^ as an “epiphyseal chondromatous giant cell tumor.” Later, Jaffe and Lichtenstein recognized it as an independent entity^2^.

Also known as Codman’s tumor^1^, this benign cartilaginous tumor usually includes an aggressive presentation, comprising 1-2% of all bone tumors and 9% of benign tumors^3^. It slightly predilects men in their second decade of life. However, up to 52% of cases often show closed growth physis^3^ ^)-(^ ^4^.

Clinically, chondroblastoma is associated with local pain (the main symptom), in common combination with synovitis and limitation of the range of motion, which has an important correlation with the typically epiphyseal location of the tumor^3^. Such location renders giant cell tumor, aneurysmal bone cyst, and osteomyelitis as the most frequent differential diagnoses^4^. Chondroblastoma also tends toward apophyseal presentations, such as in the greater tubercle of the humerus and the greater trochanter of the femur. In general, it most commonly occurs in the proximal tibia epiphysis (PTE) ^(^ ^3^ ^),(^ ^5^.

Radiographically, it manifests itself as an eccentric radioluscent image in the epiphyses of long bones, typically with well-defined margins but no halo of sclerosis. Calcifications occur often, as does aggressiveness or even pathological fractures, happening in 6.5% of manifestations. Magnetic resonance imaging studies show significant spinal cord edema in most cases^4^ ^)-(^ ^6^.

As a progressive bone tumor, most cases undergo surgical treatment, with extended curettage configuring the treatment of choice^3^ ^)-(^ ^6^. No consensus exists on the best adjuvant methods (such as argon scalpel, cryoablation, phenol, absolute alcohol, or mechanical techniques). Other methods are possible, such as radioablation for some cases and en bloc resection, which obtains a lower recurrence rate and the undesired functional worsening^7^.

The main complication consists of tumor recurrence, varying from 13.6 to 14.7% of the cases treated with curettage and a local adjuvant (according to the used surgical technique). Lung metastasis occur rarely^6^. The risk of joint degeneration, subchondral cartilage injury, and deformities stand out among the non-oncological complications due to the epiphyseal location, although few studies have observed it^4^ ^),(^ ^8^.

Due to its infrequency, relative few studies have evaluated it, some of which have described their radiographic findings and the joint complications of chondroblastoma. Moreover, this research found no prospective study on this neoplasm.

This study aims to describe the clinical and imaging characteristics of chondroblastoma to find possible factors related to local joint complications.

## METHODS

**Figure 1:**
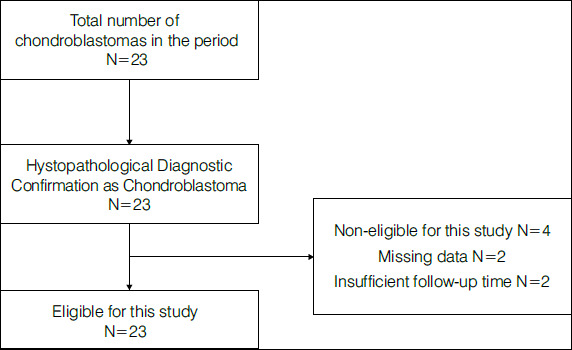
Series Stratification

Source: Prepared by the authors.

This retrospective cohort study was carried out by the Bone Tumors Group at GRAACC-IOP Hospital (Support Group for Adolescents and Children with Cancer - Institute of Pediatric Oncology); a reference service in pediatric cancer treatment to evaluate patients with chondroblastoma regarding their epidemiological and imaging profile and prognosis. The “STROBE” guideline was followed for retrospective studies to stratify the sample (Figure 1) ^(^ ^9^.

From 08/01/2009 to 12/31/2023, 23 patients diagnosed with chondroblastoma were treated at the service. During the retrospective analysis of medical records and anatomopathological reports, two patients (8.7%) failed to show the minimum data necessary for analysis, and two others (8.7%) had a minimum follow-up time shorter than 12 months. Thus, data of 19 patients were evaluated, corresponding to the series in this study.

The anatomopathological analysis of all patients was performed by the same pathologist. The imaging data of all patients were performed by the same orthopedic oncologist and a radiologist. The following variables of interest were analyzed: gender, age, follow-up time, anatomical location of the tumor, tumor dimensions, physeal plate status, presence of a sclerosis halo, calcifications, cortical rupture, spinal cord edema, edema in the adjacent joint, presence of secondary aneurysmal bone cyst, the Enneking classification^10^, presence of metastasis, presence of recurrence, treatment modality, presence of signs of joint degeneration, deformity, and the Kellgren-Lawrence classification^11^.

An analysis was carried out to determine which risk factors may be related to joint degeneration and deformities of the epiphyseal chondroblastomas. The Fisher’s exact test was used to describe the associations between categorical variables and the Student’s t-test, to compare the means of the groups of the continuous variables. The null hypothesis of this study postulated no difference between the means of the groups, in which values equal to p<0.05 were deemed statically significant (p = 0.05).

## RESULTS

**Figure 2:**
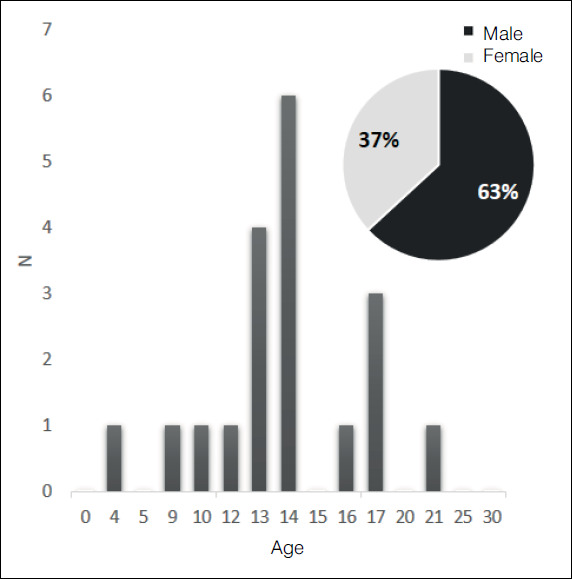
Age and gender distribution of patients suffering with chondroblastomas (n=19). The male:female gender ratio totaled 1.7:1, whereas the mean age and standard deviation, 13.6 ± 3.5 (four to 21) years.

Source: Prepared by the authors.

Our series included 19 patients with chondroblastoma, 63.2% of which were men and 36.8% women (a ratio of 1.7:1). The mean age at diagnosis totaled 13.6 years (ranging from four to 21 years) (Figure 2).

**Figure 3:**
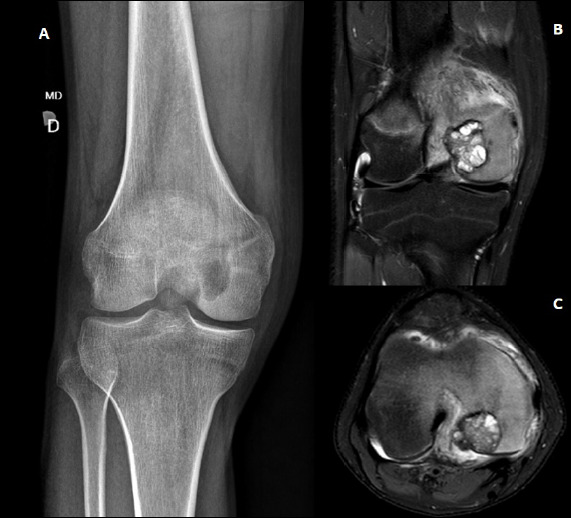
Radiography and magnetic resonance imaging of the right knee of a patient with chondroblastoma **Caption** A: Radiograph of the right knee of a 16-year-old boy with a history of moderate pain for six months. The bone lesion lies in the distal epiphysis of the femur, measuring 2.6 cm, totaling 16.7% of the total epiphysis size, showing an eccentric shape, in a closed physis and without a sclerosis halo or calcifications. B and C: Contrast-weighted magnetic resonance imaging with coronal and axial T2-weighted images showed heterogeneous signals, perilesional spinal cord edema, and cortical rupture in the region of the intercondyle in the axial section.

**Figure 4:**
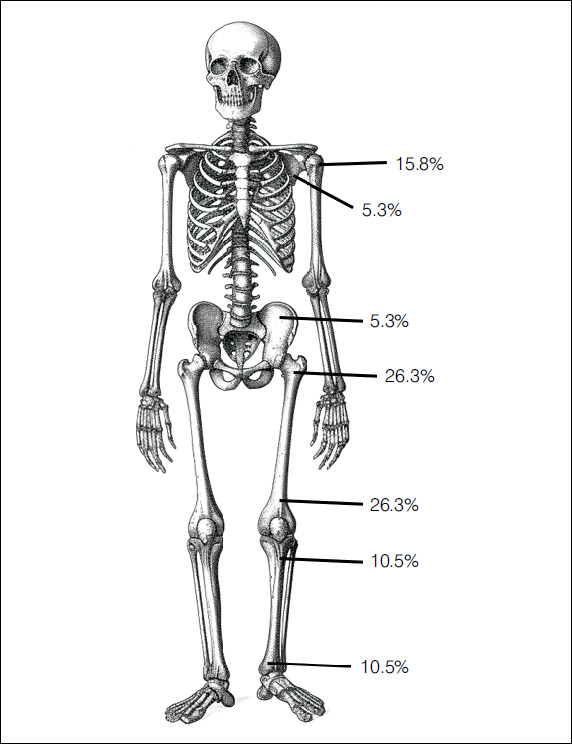
Illustration showing the distribution of the 19 cases of chondroblastoma

Source: prepared by the authors.

Injuries occurred in the lower limbs in 78.9% of patients and in their upper limbs, in 21.1% of them (4/19). The femur (10 patients, 50.0% at the distal portion and 50.0% in the proximal one), tibia (four patients, 50% at the distal portion and 50% the proximal one), and humerus suffered the most, with all three lesions affecting the proximal portion of the bone. The most affected topographies include the distal femoral epiphysis (DFE) (26.3%) (Figure 3), the greater trochanter apophysis (GTA) (21.1%), the proximal humeral epiphysis (PHE) (15.8%), and the PTE (10.5%). Figure 4 summarizes the location of the tumors.

The chondroblastomas had a medium size, measured radiographically in the anteroposterior view to total 2.87 x 3.07 mm in their laterolateral and cephalocaudal dimensions, respectively. The DFE (2.18 x 2.54 cm) and GTA (2.23 x 2.30 cm) lesions had the smallest sizes. When compared with the size of the epiphysis or the affected apophysis, the lesions occupied an average of 57.9% of the largest local diameter (24.2% in DFE, and 95.2% in GTA, with statistical significance) (p< 0.001).

**Figure 5:**
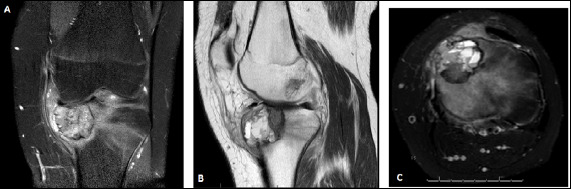
Magnetic resonance imaging of the left knee of a patient with chondroblastoma **Caption A** and **B:** Contrast-weighted magnetic resonance imaging on T2-weighted (A and C) and T1-weighted (B) sequences of the left knee of a 17-year-old boy with a history of severe pain for four months. The bone lesion lies in the proximal epiphysis of the tibia, measuring 4.0 cm, occupying 47.9% of the epiphysis and showing an eccentric shape. The coronal and sagittal sections show heterogeneous signals, perilesional spinal cord edema, synovitis in the adjacent joint, and a proximal tibial cartilage lesion. C: axial section showing rupture of the medial cortical fluid, which confirmed the secondary aneurysmal bone cyst in the anatomopathological analysis.

Of the radiographic characteristics of the lesions, 36.8% showed a centric shape and 63.2%, an eccentric one. This study considered patients’ physis status open in 3/19 of patients (15.8%), and 55.6% of the lesions failed to exceed the physeal line, whereas cortical rupture occurred in eight patients (42.1%). In total, 21.1% (4/19) of patients had secondary aneurysmal bone cysts. All lesions in the series had spinal cord edema (Figure 5). Considering the 15 epiphyseal lesions, 93.3% had associated synovitis after magnetic resonance imaging, whereas 4/15 patients (26.7%) had their damaged articular cartilage. Table 1 summarizes the imaging characteristics.

**Table 1:** Imaging characteristics of patients with chondroblastoma

Findings (%)	n=19
Centric lesion	36.8
Eccentric lesion	63.2
Open physis	15.8
Partially closed physis	31.6
Closed physis	52.6
Halo of sclerosis	42.1
Calcifications	57.9
Cortical rupture	42.1
Aneurysmal bone cyst	21.1
Bone marrow edema	100
Associated synovitis	93.3
Damaged cartilage	26.7

**Source**: prepared by the authors.

All cases received surgical treatment, one (5.3%) by en bloc resection and 18/19 (94.7%) by intralesional resection with extended curettage. Of these, 15/18 (83.3%) received autografts; one (5.5%), an allograft, and two (11.1%) (with an affected glenoid and GTA), received no filling.

Local recurrence occurred in 15.8% of cases (3/19), all of which occurred within 12 postoperative months and caused aggressive lesions according to the Enneking classification in the glenoid, PHE, and DFE. The survival time free of local recurrence at one and two years totaled 84.2%. No cases showed metastasis.

**Figure 6:**
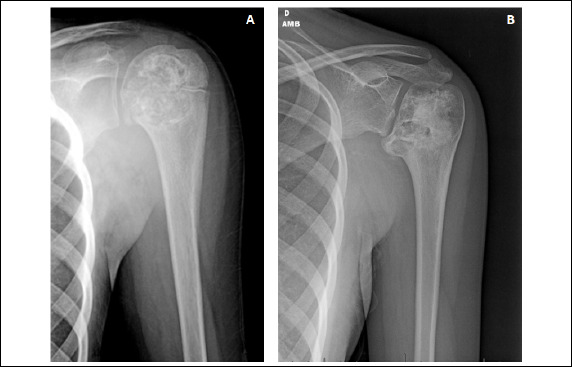
Shoulder X-ray of a patient with chondroblastoma **Caption: A:** Anteroposterior radiograph of the left shoulder of a 13-year-old girl with a history of moderate pain for a year, severe limitation of range of motion, with flexion and abduction of 30º. The bone lesion lies in the epiphysis of the proximal humerus, measuring 3.8 cm, occupying 74.0% of the epiphysis, and showing an eccentric shape, calcifications, and damaged partially closed physis. Surgical treatment was performed with curettage with a mechanical and thermal adjuvant and filling with an autologous graft of the iliac crest. B: Anteroposterior radiograph of the left shoulder four years after surgery. This study observed an important deformity of the humeral head, associated with deformity of the inferior surface of the glenoid, classified as Kellgreen-Lawrence 4.

In total, eight patients had non-oncological local complications. The most frequent complication referred to secondary arthrosis (46.7%), classified by Kellgreen-Lawrence as type 3 or 4, which occurred in only 1/5 (20%) DFE lesions. Considering only epiphyseal lesions, 5/15 (33.3%) had signs of deformity, occurring more often in the PHE (66.7%) (Figure 6).

**Table 2:** Characteristics of patients with chondroblastoma with evolution to relevant arthrosis

K/L	Age	Gender	Location	Enneking	Ratio %	2º ABC	Damaged Cartilage	Cortical Rupture	Recurrence
**3**	17	M	Proximal Tibia	3	47.9	Yes	Yes	Yes	No
**3**	14	F	Distal Femur	3	26.7	No	Yes	Yes	No
**3**	16	F	Scapula-Glenoid	3	60.2	No	Yes	Yes	Yes
**4**	13	F	Proximal Humerus	3	74.0	No	No	Yes	Yes
**4**	14	M	Distal Tibia	3	53.2	No	No	Yes	No
**4**	9	M	Proximal Femur	2	52.3	No	No	No	No
**4**	4	M	Iliac	3	95.2	Yes	No	Yes	No

1 Kellgren-Lawrence classification. ^2^ 2º ABC: Secondary Aneurysmal Bone Cyst.

## DISCUSSION

Chondroblastoma configures a rare benign tumor that can show aggressive features, including bone and lung metastasis^3^ ^),(^ ^5^ ^),(^ ^12^ ^)-(^ ^14^. Few studies have evaluated large series, and this research reports the largest series of cases observing epidemiological, imaging, and prognostic characteristics in a single Brazilian institution to date.

Results showed a prevalence of boys with a mean age of 13.6 years. The literature shows that this relation between genders always points to a higher frequency in men, despite a wide variation. Studies evince a ratio from 1.6 to 3.0, which also occurs with mean age, ranging from 11 to 25 years. However, some studies report cases of patients aged from 51 to 55 years^12^ ^)-(^ ^15^. Thus, we observed a greater tendency toward polarization of the male sex the older the sample, as in Angelini et al^13^, which considered only thane adult population with a 25-year mean age and a 3.0 gender ratio, whereas series that only considered the pediatric population showed a ratio from 1.6 to 1.8 with a mean age from 11 to 12.5 years, as in this study^14^ ^),(^ ^15^.

In this series, the most common locations refer to the proximal femur, DFE, PHE, and PTE, in agreement with much of the literature ^4^ ^),(^ ^5^ ^),(^ ^12^ ^),(^ ^14^ ^),(^ ^16^. Tumors measures a mean 3.07 cm and a 58.0% lesion-epiphysis ratio, in accordance with the literature^5^ ^),(^ ^12^ ^),(^ ^17^. A Harvard Medical School study observed that lesions larger than 5.3 cm would have a higher rate of local recurrence^12^, whereas dimensions below 2.5 cm have been the cutoff point for indicating radiofrequency radioablation as a method to treat chondroblastomas with low associated recurrence^7^ ^),(^ ^17^.

The positioning of the lesion is an important characteristic to define diagnostic hypotheses in bone tumors, in which lesions such as fibrous dysplasia, bone cysts (which typically occur centrally); and giant cell tumors (which usually manifest eccentrically) configure the classic differential diagnoses of chondroblastoma^18^ ^)-(^ ^19^. Reference textbooks for orthopedic residents show divergences, such as Campbell’s Orthopedic Surgery, which characterizes chondroblastoma as predominantly centric, and the Pediatric Orthopedics of Tachdjian, which describes chondroblastoma as eccentric^18^ ^),(^ ^19^. The sample of this study agrees with most of the literature, considering the lesion to be predominantly eccentric^3^ ^),(^ ^5^ ^),(^ ^7^ ^),(^ ^13^ ^),(^ ^16^ ^),(^ ^17^.

Formation of a sclerosis halo, presence of calcifications, cortical rupture, and cartilage violation are widely studied characteristics that, with the presence of secondary aneurysmal bone cysts, occurred from 6 to 32% of the cases in the literature, as in this study, showing no relation with the chondroblastoma prognosis ^3^ ^),(^ ^13^ ^)-(^ ^17^.

As this a lesion typically occurs in adolescents and young adults, patients’ physes were predominantly closed, a finding similar to Xu et al. ^(^ ^6^ who found 52.1% of patients with closed physes, as did Liu et al. ^(^ ^17^ ^)^ (47.2%) and Lehner et al. ^(^ ^4^ (41.6%). However, the lesion may contribute to physeal closure, despite its weak relation with anisomelia and relevant angular deformities ^3^ ^),(^ ^7^ ^),(^ ^16^.

The presence of edema on the magnetic resonance imaging shows a relevant characteristic in differentiating chondroblastomas from other diagnoses. It occurred in all cases in this study. This finding may explain the activity of the disease in our series and the fact that no lesion was classified as latent according to the Enneking classification^11^. Moreover, the resolution of perilesional edema in the follow-up of patients holds value for treatment effectiveness and clinical success^17^ ^),(^ ^18^.

Because it is a potentially aggressive lesion, the main oncological complication of chondroblastoma refers to recurrence. This occurred in a significant proportion of the cases, in agreement with other studies that considered curettage and filling as the main method of choice ^5^ ^),(^ ^13^ ^),(^ ^20^. Relapse occurs infrequently after the first year, and few studies have shown cases after two years^13^ ^),(^ ^16^. No recurrent lesion occurred in the apophyseal location or showed a sclerosis halo, secondary aneurysmal bone cyst, or completely open physis. Few studies have defined any prognostic relation between recurrence and another variable^6^ ^),(^ ^12^ ^),(^ ^14^ ^)-(^ ^16^. Wang et al. found a worse prognosis for epiphyseal versus apophyseal locations and reported that curettage of sites such as GTA showed minimal levels of technical difficulties, whereas the femoral epiphysis head showed the greatest difficulties^16^. PHE and the proximal femoral epiphysis showed the highest risk of recurrence^6^ ^),(^ ^15^. These findings were replicated in neither other studies nor in ours, which may stem from differing epidemiological profiles and sample sizes^3^ ^)-(^ ^5^ ^), (^ ^20^. All recurring lesions in this study occurred in aggressive lesions according to the Enneking classification.

Secondary arthrosis and deformities configured possible non-oncological local complications of chondroblastoma^8^ ^),(^ ^17^ ^),(^ ^20^. Farfalli et al. observed that 38% of the cases showed arthrosis, whereas Outani et al. found only 16.7%^8^ ^),(^ ^20^. Our study showed a prognostic relation between tumors classified as aggressive by Enneking and cases that evolved with relevant secondary arthrosis type 3 or 4 according to the Kellgreen-Lawrence classification (p = 0.041). Other studies have compared the same classification with anatomical location, finding better results for the DFE and worse for the proximal femoral epiphysis, PHE, and the subtalar region^8^ ^),(^ ^20^. We also observed a lower tendency of the PHE to develop joint degeneration, probably because they show smaller dimensions and joint involvement, whereas 66.7% of the lesions in the PHE evolved into arthrosis.

The main limitation of this study refers to its sample size and retrospective design, which is related to the low prevalence of chondroblastoma. Thus, its statistical analyses aim to support the findings and enable comparison of the found numbers without intending to bring a definitive answer and exhaust the subject.

## CONCLUSION

In patients with chondroblastoma, the development of secondary arthrosis showed a direct relation between the aggressiveness of the tumor according to the Enneking classification and the emergence of this complication.

Aggressiveness and clinical outcome in patients with chondroblastoma showed no causal relation when analyzed for age, sex, physeal status, or presence of calcifications or secondary aneurysmal bone cysts.
